# Partial vena cava occlusion (VCO) to counteract refractory heart failure: A new era in interventional heart failure strategy

**DOI:** 10.1016/j.amsu.2021.102387

**Published:** 2021-05-12

**Authors:** Yasar Sattar, Monil Majmundar, Talal Almas, David Song, Waqas Ullah, Homam Moussa Pacha, Mohamed Zghouzi, Islam Y. Elgendy, Fatir Murtaza, M. Chadi Alraies

**Affiliations:** a Icahn School of Medicine at Mount Sinai, Elmhurst Hospital, Queens, NY, USA; b New York Medical College, Metropolitan Hospital Center, New York, NY, USA; c Royal College of Surgeons in Ireland, Dublin, Ireland; d Abington Jefferson Health, Abington, PA, USA; e The University of Pittsburgh Medical Center, PA, USA; f Detroit Medical Center, Detroit, MI, USA; g Division of Cardiology, Weill Cornell Medicine-Qatar, Doha, Qatar; h Gomel State Medical University, Gomel, Belarus

**Keywords:** Vena cava occlusion, Heart failure with reduced ejection fraction, Preload reduction

## Abstract

**Background:**

Patients with acute decompensated heart failure are prone to recurrent exacerbation leading to poor quality of life when they do not respond to an optimal medical regimen. Due to the lack of linear positive inotropy response to increasing preload in heart failure patients, increasing preload is associated with poor outcomes. Partial occlusion of either IVC or SVC is a proposed novel treatment that can improve cardiac function or quality of life by altering preload/pressure in heart failure (HF) patients unresponsive to diuretics.

**Methods:**

PubMed, Ovid (MEDLINE), and Cochrane database we searched using the MeSH terms including “Superior vena cava occlusion,” “Inferior vena cava occlusion,” “Heart failure exacerbation.” The inclusion criteria included studies that enrolled patients > 18 years with diagnosed NYHA II-IV HF with reduced ejection fraction (HFrEF) on optimal medical treatment (OMT).

**Results:**

The analysis involved two studies with 14 patients; the mean age was 64.4 ± 10 and 100% males. The difference in the mean pulmonary pressures between pre-and-post VCO devices were 1.56 (95% CI 0.66–2.46, p-value = 0.006). There was no heterogeneity among the study of mean pulmonary pressures. With the use of VC occlusion devices, the mean difference in pulmonary artery systolic pressure decreased by 1.70 (95% CI 0.68–2.71, p-value = 0.001) (Fig. 1B). The heterogeneity of mean pressure was minimal 14%.

**Conclusion:**

In conclusion, VCO can help decrease pulmonary pressure that can indirectly prevent heart failure exacerbations and possibly hospitalization in this cohort of patients.

## Highlights

• Patients with acute decompensated heart failure are prone to recurrent exacerbation when they do not respond to an optimal medical regimen.
• Partial occlusion of either IVC or SVC is a proposed novel treatment that can improve cardiac function or quality of life by altering preload/pressure in heart failure (HF) patients.
• The choice of vena cava for device implantation is also essential. However, it is not well discussed in the literature.
• This meta-analysis aims to bridge the gap in literature that precludes the uptake of partial VCO as a routine treatment modality for refractory heart failure.

## 1 Introduction

Patients with acute decompensated heart failure are prone to recurrent exacerbation leading to poor quality of life when they do not respond to an optimal medical regimen. Preload volume is a vital contributor to a heart failure exacerbation. The increase in preload is associated with the stretching of myocardial fibers. Due to the lack of linear positive inotropy response to increasing preload in heart failure patients, increasing preload is associated with poor outcomes. Partial occlusion of either IVC or SVC is a proposed novel treatment that can improve cardiac function or quality of life by altering preload/pressure in heart failure (HF) patients unresponsive to diuretics [1,2]. This meta-analysis evaluates the degree of change in pressures with vena cava occlusion in patients with heart failure.

## 2 Methods

PubMed, Ovid (MEDLINE), and Cochrane database we searched using the MeSH terms including “Superior vena cava occlusion,” “Inferior vena cava occlusion,” “Heart failure exacerbation.” The inclusion criteria included studies that enrolled patients > 18 years with diagnosed NYHA II-IV HF with reduced ejection fraction (HFrEF) on optimal medical treatment (OMT). They underwent intermittent occlusion of IVC or SVC in artificial or natural ways. The OMT included symptomatic or mortality lowering doses of diuretics, angiotensin-converting enzyme inhibitors (ACEIs), angiotensin receptor blockers (ARBs), and beta-blockers (BBs). The primary outcome was to evaluate the change in pulmonary artery pressure as an indirect means of lowering the worsening of heart failure exacerbations.

## 3 Results

**Fig. 1:**
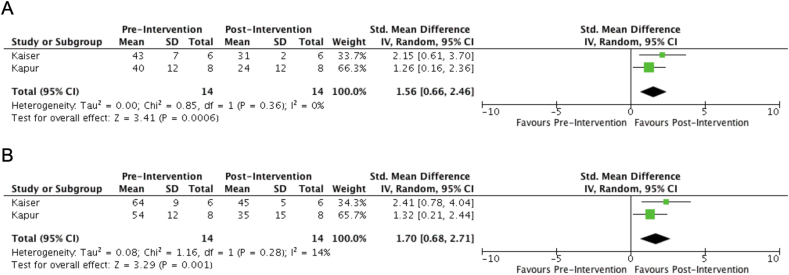
A) Showing mean pulmonary pressures pre- and post-vena cava occlusion device. B) Showing mean pulmonary artery systolic pressures.

The analysis involved two studies with 14 patients; the mean age was 64.4 ± 10 and 100% males. The difference in the mean pulmonary pressures between pre-and-post VCO devices were 1.56 (95% CI 0.66–2.46, p-value = 0.006) (Fig. 1A). There was no heterogeneity among the study of mean pulmonary pressures. With the use of VC occlusion devices, the mean difference in pulmonary artery systolic pressure decreased by 1.70 (95% CI 0.68–2.71, p-value = 0.001) (Fig. 1B). The heterogeneity of mean pressure was minimal 14%.

Safety measures and adverse effects of IVC and SVC occlusion using preCARDIA catheter were discussed in Kapur et al. Acute IVC occlusion reduced left ventricular systolic and diastolic pressures, LV volumes, cardiac output (CO), and systemic blood pressure; in contrast, SVC occlusion reduced LV diastolic pressure and volumes without affecting cardiac output or systemic blood pressure. SVC therapy using preCARDIA was well tolerated with stable MAP and CO without evidence of damage to SVC, lungs, heart, or brain. However, in one animal study, spontaneous sinus bradycardia and hypotension were observed after 14 hours of SVC therapy, but no damage to SVC, RA, RV, brain, or lungs was noted. A large anterior wall myocardial infarction due to LAD ischemia and reperfusion injury was also documented at autopsy. However, in all three animal studies in Kapur et al., SVC occlusion increased IJ pressure, reduced RA, mean PA, PCWP pressure; CO remained stable. One potential concern is the impact of elevated jugular venous pressure on the cerebral function, but no neurologic deficits were identified during or up to 7 days of follow-up after SVC occlusion [1].

Quality assessment was done as per the “Cochrane systemic reviews” method^3^. The quality of the studies was moderate—Table 1 and Table 2. Readmissions with acute decompensated heart failure remains a concern among patients with HFrEF [3,4].

**Table 1:** Baseline and procedural characteristics of the included studies.

Study	No of patients	Male	Age	NYHA class	LV EF	LV end diastolic diameter	Inotropes	Diuretics	Creatinine	SVC diameter mm	PA systolic pressure pre-occlusion	PA systolic pressure post-occlusion	PA mean pressure pre-occlusion	PA mean pressure post-occlusion
Kaiser 2019	6	6	69 ± 3.4	–	–	–	–	–	–	–	64 ± 9	45 ± 5	43 ± 7	31 ± 2
Kapur 2019	8	8	61 ± 6	3.6 ± 0.5	19 ± 10	5.9 ± 1.1	2	8	1.5 ± 0.7	24 ± 3	54 ± 12	35 ± 15	40 ± 12	24 ± 12

**Table 2:** Quality of Cross-Over Studies Involved in Cochrane Systematic Reviews. B. Quality assessment of the studies as per the modified tool for quality assessment for case series.

Study	Appropriate cross over design	Randomized treatment	Carry over effect	Unbiased data	Allocation concealment	Blinding	Incomplete outcome data	Selective outcome reporting	Other bias
Kaiser 2019	Low	Low	Low	Low	Low	Low	Low	Low	unclear
Kapur 2019	Low	Unclear	Low	Low	Low	High	Low	Low	Unclear

## 4 Discussion

The non-invasive inferior vena cava occlusion by exercise is reviewed by Kaiser et al. enrolled six patients that underwent particle occlusion of IVC by exercise. The study found that mean pulmonary artery pressures dropped significantly with occlusion with no additional neurological or cardiovascular complications. The study found that IVC obstruction lowers left ventricular filling pressure, decreasing cardiac workload, and improving myocardial contractility [1]. Another study by Kapur et al. used an SVC occlusion device called Precardia to occlude the SVC [2] intermittently. Kapur et al. involved eight patients with HFrEF. All patients underwent Precardia device SVC intermittent occlusion. In this study, at 5 minutes follow-up, JVP promptly rose and returned to baseline after cessation of the occlusion. While the occlusion significantly reduced the atrial and ventricular end-diastolic pressures in all the patients, there was no change in mean arterial pressure (MAP) and CO of these patients.

Additionally, none of the patients reported any adverse neurological or cardiovascular sequelae immediately after the procedure. The potential concern is the impact of elevated jugular venous pressure. However, after 1 week, follow-up divulged that none of the patients developed cerebrovascular accident, myocardial infarction, neurologic complications of thrombosis secondary to the procedure, demonstrating a very low risk of grave post-procedural outcomes.

The results delineated by Kapur et al. depicted that partial VCO can be a safe therapeutic procedure that can be efficaciously employed in heart failure patients. The Kapur et al. study results were significant and got expedited approval of the device by the USA's food and drug administration (FDA) [5]. These studies demonstrated that partial reduction of left ventricular volume by reducing preload from invasive or non-invasive VCO might have several advantages. Firstly, sustained increased cardiac contractility potentially increases cardiac output. Secondly, VCO reduces right ventricular pressure and volume overload that can shift the interventricular septum towards the right ventricle, increasing the LV capacitance and stroke volume [6]. Thirdly, elevated central venous pressure is associated with systemic congestion associated with impaired renal function and diuretic resistance [7]. Hence, correcting systemic venous congestion promotes renal function and improves diuretic responsiveness, thereby leading to earlier decongestion of patients with acute decompensated heart failure. To achieve these benefits for a prolonged period, we need an intermittent device occluding the vena cava. The choice of vena cava for device implantation is also essential. However, it is not well discussed in the literature. The study by Kapur et al. experimented in superior vena cava. Cerebral hypoperfusion is a concern regarding SVC occlusion. Although there was no case of neurological impairment in the study by Kapur et al., the study included only eight patients that are not adequate to assess the safety.

On the other hand, the device implantation in Inferior vena cava precludes two-third of blood supply and might lead to profound hypotension. It also increases pressure in the splanchnic and renal vein that may lead to renal injury and reduces oral medications' absorption. Still, more studies are needed in this therapeutic strategy since it is a relatively novel approach.

Our study analyzed both studies to see the mean pressure difference as an indirect effect on pulmonary congestion. The mean difference of pulmonary pressures and mean systolic pressures between pre-and-post VCO were 1.56 (95% CI 0.66–2.46, p-value = 0.006) and 1.70 (95% CI 0.68–2.71, p-value = 0.001), respectively. Given the drop of 1.70 mmHg in PASP, mild clinical improvement in pulmonary hypertension, the results are limited to see the long-term effect of this change. Nevertheless, the study has several limitations. Kaiser et al. was published as abstract, so baseline characteristics are not available. The power of the study is very low for the generalization of results. The long-term outcome of the procedure is yet to be evaluated, and the studies had a very short follow up period. Since the study has shown promising preclinical results, more randomized trials with adequate follow-up are needed to evaluate the treatment option better.

## 5 Conclusion

In conclusion, VCO can help decrease pulmonary pressure that can indirectly prevent heart failure exacerbations and possibly hospitalization in this cohort of patients. Therefore, in patients with refractory heart failure unresponsive to appropriate medical intervention, partial VCO might be the way forward.

## Ethical approval

NA.

## Sources of funding

NA.

## Author contribution

YS, MM, TA: conceived the idea, designed the study, and drafted the manuscript.

DS, WU, HMP: conducted literature search and created the illustrations.

MZ, IYE FM,: revised the manuscript critically and refined the illustrations.

YS, MCA revised the final version of the manuscript critically and gave the final approval.

## Consent

NA

## Registration of Research Studies

Name of the registry: NA.

Unique Identifying number or registration ID: NA.

Hyperlink to your specific registration (must be publicly accessible and will be checked): NA.

## Guarantor

Talal Almas.

RCSI University of Medicine and Health Sciences.

123 St. Stephen’s Green.

Dublin 2, Ireland.

Talalalmas.almas@gmail.com

+353834212442.

## Provenance and peer review

Not commissioned, externally peer reviewed.

## Disclosure

None.
