# Supplementary material for: Partial vena cava occlusion (VCO) to counteract refractory heart failure: A new era in interventional heart failure strategy
Source: Ann Med Surg (Lond). 2021 May 12;66:102387. doi: 10.1016/j.amsu.2021.102387 (PMC8141653; doi:10.1016/j.amsu.2021.102387)
Supplement: Multimedia component 2 [file mmc2.docx]

**AMSTAR 2 CHECK LIST**

**Box 1 AMSTAR 2 critical domains**

- $\surd$Protocol registered before commencement of the review (item 2)
- $\surd$Justification for excluding individual studies (item 7)
- $\surd$of bias from individual studies being included in the review (item 9)
- $\surd$Appropriateness of meta-analytical methods (item 11)
- $\surd$Consideration of risk of bias when interpreting the results of the review (item 13)
- $\surd$Assessment of presence and likely impact of publication bias (item 15)

#### Box 2 Rating overall confidence in the results of the review

- $\surd$**High**
- No or one non-critical weakness: the systematic review provides an accurate and comprehensive summary of the results of the available studies that address the question of interest
- **Moderate**
- More than one non-critical weakness*: the systematic review has more than one weakness but no critical flaws. It may provide an accurate summary of the results of the available studies that were included in the review
- **Low**
- One critical flaw with or without non-critical weaknesses: the review has a critical flaw and may not provide an accurate and comprehensive summary of the available studies that address the question of interest
- **Critically low**
- More than one critical flaw with or without non-critical weaknesses: the review has more than one critical flaw and should not be relied on to provide an accurate and comprehensive summary of the available studies
- *Multiple non-critical weaknesses may diminish confidence in the review and it may be appropriate to move the overall appraisal down from moderate to low confidence
